# Bessel Beam: Significance and Applications—A Progressive Review

**DOI:** 10.3390/mi11110997

**Published:** 2020-11-11

**Authors:** Svetlana Nikolaevna Khonina, Nikolay Lvovich Kazanskiy, Sergey Vladimirovich Karpeev, Muhammad Ali Butt

**Affiliations:** 1 Department of Technical Cybernetics, Samara National Research University, 443086 Samara, Russia; khonina@ipsiras.ru (S.N.K.); kazanskiy@ipsiras.ru (N.L.K.); karp@smr.ru (S.V.K.); 2 IPSI RAS-Branch of the FSRC “Crystallography and Photonics” RAS, 443001 Samara, Russia; 3 Institute of Microelectronics and Optoelectronics, Warsaw University of Technology, Koszykowa 75, 00-662 Warszawa, Poland

**Keywords:** optical trapping, material processing, free-space long-distance self-healing beams, optical coherence tomography, superresolution, sharp focusing and polarization transformation, depth of focus

## Abstract

Diffraction is a phenomenon related to the wave nature of light and arises when a propagating wave comes across an obstacle. Consequently, the wave can be transformed in amplitude or phase and diffraction occurs. Those parts of the wavefront avoiding an obstacle form a diffraction pattern after interfering with each other. In this review paper, we have discussed the topic of non-diffractive beams, explicitly Bessel beams. Such beams provide some resistance to diffraction and hence are hypothetically a phenomenal alternate to Gaussian beams in several circumstances. Several outstanding applications are coined to Bessel beams and have been employed in commercial applications. We have discussed several hot applications based on these magnificent beams such as optical trapping, material processing, free-space long-distance self-healing beams, optical coherence tomography, superresolution, sharp focusing, polarization transformation, increased depth of focus, birefringence detection based on astigmatic transformed BB and encryption in optical communication. According to our knowledge, each topic presented in this review is justifiably explained.

## 1. Introduction

**Figure 1:**
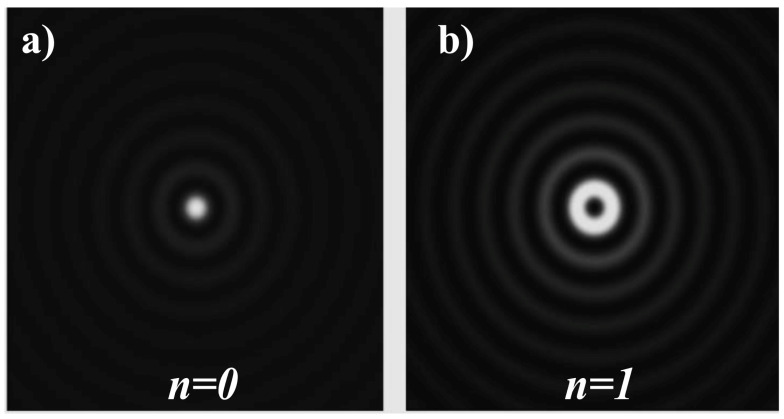
(**a**) Transverse profile of an ideal BB intensity (square of the zero-order Bessel function), (**b**) Transverse profile of the intensity of the first-order BB.

Bessel functions are precise solutions to the Helmholtz equation [1,2], i.e., classic Bessel beams (BB) are diffraction free and offer noteworthy features such as an exceptional depth-of-field (DOF), self-recovery, and beam-width relative to the scattering limit. Moreover, high-order BBs show phase displacements, i.e., they imitate vortex beams and transfer an orbital angular momentum [3]. BB modes are characterized by an integer *n*, known as the order of the beam. In cylindrical coordinates, the complex amplitude for the *n*th-order BB can be expressed as:

(1) $$E_{n}(r,\varphi ,z)=Ae^{ik_{z}z}J_{n}(k_{r}r)e^{\pm in\varphi }$$

where *A* is the complex constant, $k_{z}=k\cos\theta_{0}$, $k_{r}=k\sin\theta_{0}$. k=2π/λ is the wavenumber and *λ* is the wavelength of the light radiation, $\theta_{0}$ is the angle of the conic wave to the z-axis. The function *J_n_ (x)* is the *n*th-order Bessel function of the first kind. The zero-order beam *E*_0_ has a maximum intensity on the axis, similar to the Gaussian beam (GB); but, unlike the GB, it also has a collection of circular nodes ringing the axis as seen in Figure 1 [4]. But there is also a minimum on the central axis for *n* ≥ 1. The on-axis node occurs for the same reason as in the case of Laguerre–Gauss case: the $e^{\pm in\varphi }$ component means that the beam has orbital angular momentum (OAM) *L_z_* = *nħ* along the beam axis. As a consequence, the phase is unknown or singular on the axis itself, and thus the intensity must disappear. It is difficult to construct a true BB in the lab because it would have to have infinite reach in the transverse direction, and it would have infinite power. In this respect, it is identical to a true plane wave, which also has an infinite amplitude and thus can never be realized precisely. But as with the plane wave, it is possible to generate very good approximate BBs, which have all the useful properties of the idealized version, at least for some finite area. It can be seen that all bright rings hold the same amount of power; thus, for a beam of finite power, the intensity of each ring must decline as the number of rings increases. The strength of the light in the central core must also reduce as the number of rings increases. The most effective technique for producing an approximation to a zeroth-order BB is utilizing an axicon, both refractive (conically shaped) and diffractive versions of optical elements. When illuminated by a GB with a waist size much smaller than the hard aperture of the axicon, virtually the whole input intensity is converted into a BB. The spacing between the rings of the BB depends on the period of the axicon. Usually, axicons have been used to generate zeroth-order BBs. Higher-order BBs can be directly generated from an illuminating GB by use of axicon-type computer-generated holograms, for example, diffractive spiral axicons [5,6].

In 1987, BBs were first studied by Durnin [1] and have been widely employed in applications related to both optics [7,8] and acoustics [9,10,11]. In acoustics, BBs are generally used in applications such as ultrasound imaging systems [12,13,14]. Their extended DOF and slender beam-width provide a precise scanning of the transmitted beam, whereas their self-recovering properties contribute toward extraordinary robustness to tissue scattering. Moreover, its diffraction-free feature provides a perpetual deep imaging resolution. The diffraction-free self-healing features of the BB permits it to penetrate deep into the volume of a sample, resist against refractions in chaotic environments, and provides an axial resolution as compared to that of GBs. The fields, formed from coherent mixtures of BBs, reveal a more than ten-fold rise in their undistorted penetration to diffraction-limited beams. Recently, vortex beams, to be specific, BBs, have gathered a lot of interest because of their distinctive properties for particle trapping [15,16,17,18], particle handling/rotation applications [19,20,21] or acoustic radiation force strategies in liquids [22]. BBs have also been employed in photopolymerization [23] as well as in material processing [24]. However, their prospects for high-throughput 3D printing have not been effusively investigated.

**Figure 2:**
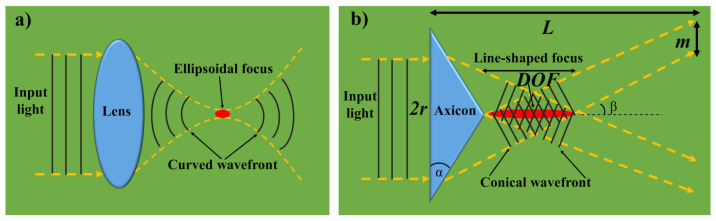
An illustration associating Gaussian and BB; (**a**) The incoming Gaussian laser beam is focused by a lens to form an ellipsoidal focus by transforming plane waves into a curved wavefront; (**b**) A BB is generated by an axicon which consists of a conical wavefront and a central lobe creates a focus which resembles a line-shape.

In general, laser beams have a Gaussian profile. When a GB is focused via an optical lens, an ellipsoidal focus is formed as revealed in Figure 2a, which is described by a lateral spot size and a confocal length. A perfect BB is produced by a congregating conical wavefront of an unbounded level. When it congregates to the axis of symmetry and interacts with itself, the slanted conical wavefront creates the distinctive BB shape as displayed in Figure 2b. BBs are expressed as a single-ring pattern emerging from their narrow angular spectrum in the far-field. Although a true BB would involve an infinite amount of energy to create, an axicon produces a close approximation with nearly non-diffracting properties within the Axicon’s depth of focus (*DOF*). *DOF* is a function of the radius of the beam entering the axicon (*r*), the axicon’s index of refraction (*n*), and the alpha angle (*α*):

(2) $$DOF=\frac{r\sqrt{1-n^{2}\sin^{2}\alpha }}{\sin\alpha \cos\alpha (n\cos\alpha -\sqrt{1-n^{2}\sin^{2}\alpha })}\approx \frac{r}{(n-1)\alpha }$$

The basic equation assumes that the angle of refraction is small and becomes less accurate as *α* decreases. Beyond the axicon’s *DOF*, a ring of light is produced. The thickness of the ring (*m*) remains constant and is comparable to *r*:

(3) $$m=\frac{r\sqrt{1-n^{2}\sin^{2}\alpha }}{\cos\alpha (n\sin^{2}\alpha +\cos\alpha \sqrt{1-n^{2}\sin^{2}\alpha })}\approx r$$

Along with the numerous benefits of the BBs, they have their drawbacks as well. Firstly, these are energy characteristics: diffraction-free properties are achieved due to the high energy cost. The axial length of the beam is accompanied by a decrease in the amount of energy on the optical axis because only a small part of the incident energy (energy from separate rings) is concentrated in the central spot. Secondly, the presence of peripheral rings: a decrease in the size of the focal spot is accompanied by the growth of side lobes that worsen the image properties [25,26]. Likewise, the high sensitivity of the axicon’s point spread function (PSF) to aberrations of the optical system is known [27,28]. However, in microscopes and scanning optical systems, the side lobes with an intensity smaller than 30% of the main light spot can be efficiently filtered out [29,30]. This possibility is implied in Section 5.

Two-photon microscopy has revolutionized functional cellular imagining in tissue, but the highly confined DOF of standard set up yields great optical sectioning, it also restricts the speed of imagining in volume samples and user-friendliness. Therefore, a basic and overhaul adjustment to the two-photon laser scanning microscope has recently been seen, which expands the DOF by the use of an axicon. Here, three major advantages of this method are demonstrated in [31] using biological samples widely used in the neuroscience field. First, a sample of neurons grown in culture and moved along the z-axis displays that a steady focus is attained without negotiation on the transverse resolution. Secondly, in an acute slice of the living mouse cortex, 3D population dynamics are monitored which demonstrates that faster volumetric scans can be performed. Thirdly, in a fixed sample of mouse cortex, a stereoscopic image of neurons and their dendrites are obtained via two scans instead of the full stack required by the standard systems. These benefits make extended DOF imagining based on BBs highly suitable for the field of microscopy and life sciences in general, together with the ease of incorporation into pre-existing systems.

Non-diffractive beams play a special role in fluorescence microscopy based on light sheets (planar microscopy) [32,33,34,35,36]. Light-sheet fluorescence microscopy (LSFM) provides extremely high image processing speed, good signal-to-noise ratio, a low level of photo-bleaching, and good optical depth of penetration. This unique combination allows you to successfully apply this technology to the study of living microorganisms in real-time. The extremely low toxicity of the system allows the ability to view and differentiate groups of cells without causing damage to the samples. Such a system is specifically designed for research in marine and cellular biology, as well as in plant physiology.

In acoustics, there are several approaches to produce zeroth and high-order BBs, though it is not possible to perfectly generate all of them in reality. Numerous techniques are suggested to create truncated BBs. A straightforward and attractive scheme is to employ a spherical slit at the axis, as a result, BB patterns are produced, in equivalence with the optics [2]. Nevertheless, the tiny opening intensely restricts the transmitted energy. Several works [37,38,39,40] have been published on well-generated BBs employing a periodic arrangement of circular active piezoelectric sources in such a way that every circular active element is fixed conferring to a Bessel function in both phase and amplitude.

There is a striking resemblance between the radial modes of piezoelectric sources and the Bessel function, which is why this phenomenon can be imitated employing simple discs. However, it will not provide a very high radiation efficiency [41]. A little while ago, it was assumed that BBs can only be produced via vastly coherent light sources, for example, solid-state and gas lasers. However, over the last few years, several works have described different techniques to produce BBs employing different light sources such as semiconductor light sources [42], vertical-cavity surface-emitting lasers [43,44], light-emitting diodes [43] and vertical external-cavity surface-emitting lasers [45], among others.

**Figure 3:**
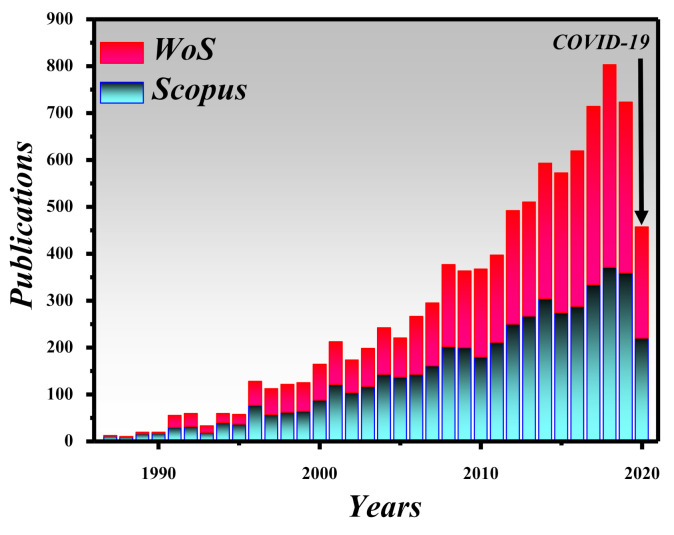
Articles related to “BBs” indexed in the Scopus database and Web of Science published during the period 1987–2020.

The topic of the BB is very interesting and has been under consideration by several researchers around the world. For instance, the number of publications on the “BB” topic between 1987 and 2020 is depicted in Figure 3. This graph shows the number of papers indexed in the Scopus and Web of Science databases, which are considered to be the most reliable and authentic sources. It can be seen that BB has a constantly increasing trend in the number of articles published each year, similar to other optics or physics-related topics. However, in 2020, we can see fewer publications on BBs, mainly due to COVID-19 which has decelerated research activities around the globe. There are several other indexing systems such as Copernicus, Science Citation Index, Google Scholar and PubMed, among others.

In this review paper, we have briefly explained the methods for the generation of BBs and recent advances in the utilization of these extraordinary beams in several applications such as optical trapping, material processing, self-healing property for underwater optical communication, optical coherence tomography, superresolution, sharp focusing and polarization transformation, increased depth of focus and other related applications. Over the last few years, our research group at the department of Technical Cybernetic, Samara National Research University, Russia has published several papers on the exploration of different applications utilizing BBs, such as optical trapping [46,47,48,49], material processing [50,51,52,53,54], free-space long-distance self-healing [55,56,57,58], astigmatic transformed BBs [59,60,61], encryption in optical communication [62,63,64,65] and sharp focusing [66,67,68,69,70], among others.

## 2. Optical Trapping with BBs

The techniques of tweezing and optical trapping are established on the forces that emerge as a result of the rule of momentum preservation in the reflection, refraction, and absorption of the laser beam at the particle [71,72]. For effective optical handling, i.e., consistent microparticles trapping, a high gradient of the optical power density is required. For instance, a sharply focused laser light beam is essential to trap the microparticle. This considerably shrinks the working area owing to diffraction, tighter focusing outcomes in faster radiation divergence, and the issue cannot be solved regarding the Gaussian optics. Nevertheless, Durnin [1] has demonstrated in his remarkable work that the diffractive divergence can be practically removed with a special class of non-diffracting light fields known as BBs. Practically speaking, BBs display a limited propagation length, which is dependent on the width of the original collimated beam. As the BBs preserve their focus throughout the way, the placement of the manipulated object can be considerably altered, hence allowing practical flexible micromanipulation systems. Additionally, the employment of BBs unveils novel prospects in micro-fabrication [73], manipulation of micro-machines [74] and numerous “lab-on-a-chip” applications [75].

Optical traps based on focused BBs offer highly superior capabilities for manipulation of individual glass beads in the three spatial directions in comparison with standard optical tweezers based on focused GB [76]. Interesting possibilities for the simultaneous trapping and rotating of various types of dielectric particles (with high and low refractive indexes) provide multi-ringed vortex beams [77]. The angular momentum density of BBs is similar to that of multi-ringed LG beams [78,79]. However, high-order BBs are easier to generate experimentally [46].

The field of optical trapping has grown into an essential phenomenon in the biological examination, cold atom physics, and genetics [80,81]. It has swiftly bee established from its first verification by Ashkin in the 1970s [71]. Regardless of its extensive implementation, the optical trapping of airborne particles employing a single-beam gradient-force was not verified until 1997 [82]. Even nowadays, most of the laser tweezer operations are being used to trap fairly translucent specks in a liquid. A particle trapping via a single laser beam in the air is a more challenging task than in a liquid medium, as the optical trap should be robust to conquer gravity and disruption in the air. Moreover, as opposed to a particle in a liquid medium, a particle in the air has a higher relative refractive index, which induces a sturdy scattering force that tends to disrupt the trap [83]. Nevertheless, for particle delivery strategies, the potentiality to trap airborne particles is essential [84] as well as developing aerosol classification methods that incorporate optical trapping with examination practices, for instance, Raman spectroscopy [83,85].

Consequently, significant attention has been given to develop novel methods to capture airborne particles utilizing either the photophoretic force or the radiative pressure force. In recent times, the photophoretic force has been used to permit optical trapping of airborne particles with absorbing properties. Several beam shapes such as vortex beams [86], bottle beams [87], and hollow cones produced via a single beam [88], tapered rings [89], two counter-propagating beams [90] and optical lattices [91], among others, have been employed. In [86,87,88,89,90], the particles are trapped in the low-intensity area, which is contrasted to laser tweezers where the gradient force traps the particles near a laser beam’s high-intensity focal point. As several photophoretic traps are only successful for particles with a specific geometry [92], the morphology of the absorbing particles poses additional technical hitches. Nevertheless, some applications include the ability to capture the particles irrespective of their construction and absorption.

**Figure 4:**
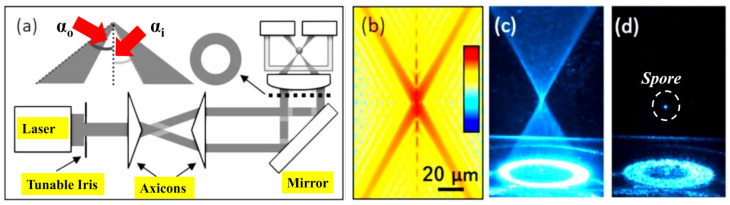
(**a**) Illustration of the optical trapping setup. The diameter of an expanded laser beam is managed with a tunable iris before travelling through the two axicons to form a collimated hollow beam. The aspheric lens creates a hollow conical focus within a glass chamber where airborne particles are trapped, adapted with permission from [93]. (**b**) Calculated intensity profile near the focal spot, adapted with permission from [93]. (**c**) Image of the conical focal region produced inside the chamber, adapted with permission from [93]. (**d**) Image of a spore captured in the air close to the focal point, adapted with permission from [93].

An interesting method is proposed in [93], where a single formed laser beam is used to form a region of low light intensity for photophoretic trapping of absorbing particles but minimizing the dispersion force near the focal point at the same time. As a result, radiative pressure found that the trapping of translucent particles is facilitated as shown in Figure 4a–d. Besides, this low dispersion force permits airborne particles to be trapped by radiative pressure-based trapping via relatively low numerical aperture optics. A comparable method has lately been demonstrated to allow optical trapping of high relative refractive index non-absorbing particles utilized to trap airborne aerosol droplets [94].

Due to the transfer of momentum from photons to particles, the radiative pressure force, the gradient force and the scattering force produced from a single laser beam emerges. Regardless of the realization of optical tweezers in maneuvering particles in the fluid, optical traps based on radiative pressure utilizing a single laser beam are still not suitable for grabbing airborne particles. Bearing in mind the radiative pressure force as an amalgamation of a gradient force and a scattering force, this problem can be solved. The gradient force drags a particle close to the focus of the laser beam into the high-intensity region at the focus, ensuring the restoring force is mandatory to catch a particle. Instead, the scattering force moves the particle in the direction of the propagation of light and off-limits the reinstating force needed. In general, optical trapping is only probable when the gradient force overpowers the dispersion force [82]. Airborne particle trapping is a challenging task due to the high refractive index of a particle in the air comparative to the ambient medium results in a sturdy scattering force. To form a robust gradient force to trap airborne particles, high numerical aperture optics (typically NA > 0.9) are obligatory [82]. In principle, by utilizing a counter-propagating beam configuration, the scattering force can be concealed [95]. However, an accurate orientation is required, which is practically not possible in many applications. If a laser light falls on absorbing airborne particles, a portion of the light is absorbed and transformed into heat. Consequently, an interplay between a non-uniformly heat-emitting particle and the ambient gas molecules leads to the photophoretic force. As a result, absorbing airborne particles are captured by the photophoretic force. For instance, if an absorbing airborne particle is hit by a light from one side, due to impact with the hot part of the particle, the gas molecules on the elevated temperature side of the particle will have greater velocities, imposing a net force that moves the particle to its cold side. The photophoretic force can be ~5 orders of magnitude more robust than the gradient force for an intensely absorbing particle, usually employed in optical tweezers [96].

## 3. Material Processing via Ultrafast BBs

These days, micro- and nano-technologies rely on the growth of detailed and manageable manufacturing tools that are capable of structuring materials with full precision and minimum collateral loss. In a range of applications, from entertainment to medicine, lasers have proven to be flexible instruments over the years. The individual usage of this system is dependent on different parameters of the radiation emitted, such as wavelength, energy, and pulse duration. When considering some applications, the beam structure is essential [97,98,99]. A typical GB is appropriate for some of them. The ultra-short laser processing technique has grown into a vital technology capable of delivering fundamental processing skills well into the size of the nanoscale [100,101,102]. This counts on incomparable material processing capabilities through nonlinear excitation and inadequate thermal diffusion, resulting in high-end applications where energy localization in space and time by ultrashort pulses is important. Novel models of laser material processing are developed based on the spatiotemporal design of irradiation rendering to the material response to optimize the structuring process concerning quality and scale [103,104,105]. To synergistically associate irradiation and material reaction to energy load, an advanced processing strategy involves a detailed understanding of the irradiation and material transformation method.

For material processing applications in ultrafast modes, a new class of ultrafast laser beams has recently emerged with the potential to achieve processing precision beyond the diffraction limit, deep into the nanoscale domain. This relies on non-diffractive concepts, especially the Bessel-Gauss beam [106], where non-diffractive propagation can be used to design the multi-dimensional interaction segment. In transparent materials, the ability of these Bessel-Gauss beams is entirely utilizable, with an energy gap larger than photon energy. BBs are helpful in Bragg grating inscription [107], microchannel forming [108] and photopolymerization [109] due to an elongated focal area. BBs have a ring-shaped spatial spectrum. It is conceivable to attain Mathieu beams with elliptical intensity distribution by modifying the phase and amplitude of the BB. For instance, the elliptical beam structure has been shown to cause directional glass cracking. Zeroth and higher-order BBs are among the most distinguished non-diffracting beams and can be comprehended utilizing diffractive holograms or by focusing Laguerre GBs via conical prisms (axicons).

In [110], a novel method is developed for the realization of superimposed complex BB vortices of different orders, single and superimposed Mathieu beams of different topologies, as well as parabolic non-diffracting beams. Furthermore, their amplitudes and phases are controlled in the focal region independently to assist the creation of complex patterns not only in the transverse plane but also along the focal line. The experimental verification using a conical prism together with a geometrical phase element is presented for the transparent materials. In [111] the higher-order vector BBs are produced for transparent material processing applications.

Three-dimensional integrated circuits are an enticing option to replace standard two-dimensional ones as high-efficiency, low power exhaustion and miniaturized foot-print microelectronic devices [112]. One of the primary enduring problems, however, is the processing of high-aspect-ratio through-silicon vias (TSVs), which is a critical technology for the assembly of three-dimensional silicon integrated circuits. As a simple and environmentally friendly substitute with less manufacturing stages due to the exclusion of photolithography, TSV manufacturing through direct laser drilling has been suggested. This has been demonstrated with the production of 20 μm diameter holes in a 250 μm thick silicon substrate employing nanosecond UV laser percussion [113]. However, for the assembly of three-dimensional silicon integrated circuits, the deep drilling of holes smaller than 10 μm in diameter remains a major future challenge. Ultrafast laser treatment has recently been shown to be a desirable method for material processing, as it facilitates sub-diffraction-limit processing with heat-affected zone elimination [114]. High-quality cutting has been successfully checked for planar silicon elements with 780 nm femtosecond lasers [115]. Furthermore, in many important research and engineering applications, fast speed and high aspect ratio drilling of through-holes in different materials utilizing ultrafast laser processing have become an attention-grabbing theme. In different silica glasses, femtosecond BBs have been employed to create microstructures with aspect ratios of up to 10^2^–10^3^, without requiring sample transformation due to the elongated field depth.

**Figure 5:**
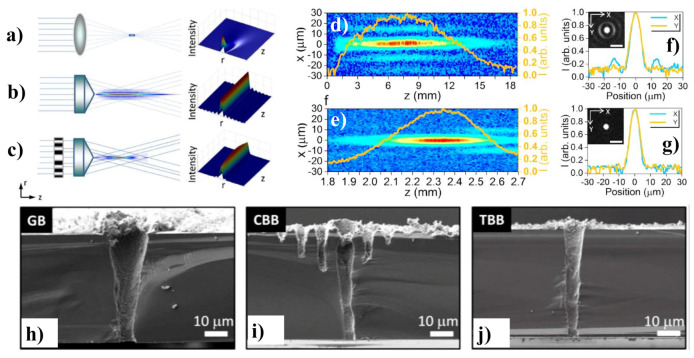
Diagram of the beams generation setup and intensity profiles for, (**a**) GB, (**b**) CBB and (**c**) TBB. Experimentally measured two-dimensional profiles in the x-y plane in cooperation with the on-axis intensity distribution (Orange curve) of, (**d**) CBB and (**e**) TBB. Measured transverse intensity distributions at *z = z_max_* along the CCD captured images of (**f**) CBB and (**g**) TBB. SEM images of the cross-sectional view of TSVs manufactured silicon substrate using (**h**) GB, (**i**) CBB and (**j**) TBB. Adapted with permission from [117].

In [116], TSVs are fabricated by using femtosecond BBs with wavelength tuning of 400 nm to 2400 nm. The manufacture of fine TSVs utilizing a 1.5 μm femtosecond BB is demonstrated in [117]. A Bessel femtosecond beam is customized via a specifically fabricated binary phase plate to remove the extreme ablation brought by the sidelobes of a traditional BB. Three separate forms of femtosecond laser beams such as a Gaussian beam (GB), a conventional BB (CBB) and a tailored BB (TBB) were used to determine the theory of the suggested method as shown in Figure 5a–c. It is established that the customized femtosecond BB (without sidelobe destruction) can be employed to form a two-dimensional periodic arrangement of ~10 μm TSVs on a silicon substrate of thickness 100 μm, indicating a possible use in the three-dimensional assembly of three-dimensional silicon integrated circuits. Two-dimensional profiles of CBB and TBB in the x-y plane together with the on-axis intensity distributions (orange curve) are experimentally measured as displayed in Figure 5d,e, respectively. The experimentally obtained transverse intensity distributions of the CBB and TBB at *z = z_max_* are presented in Figure 5f,g, respectively, together with a charged coupled device taken pictures of the particular beams in the insets. Figure 5h–j illustrate the SEM pictures of the TSVs formed in 50 μm thick samples employing the GB, CBB and TBB, respectively.

## 4. Free-Space Long-Distance Self-Healing BBs

**Figure 6:**
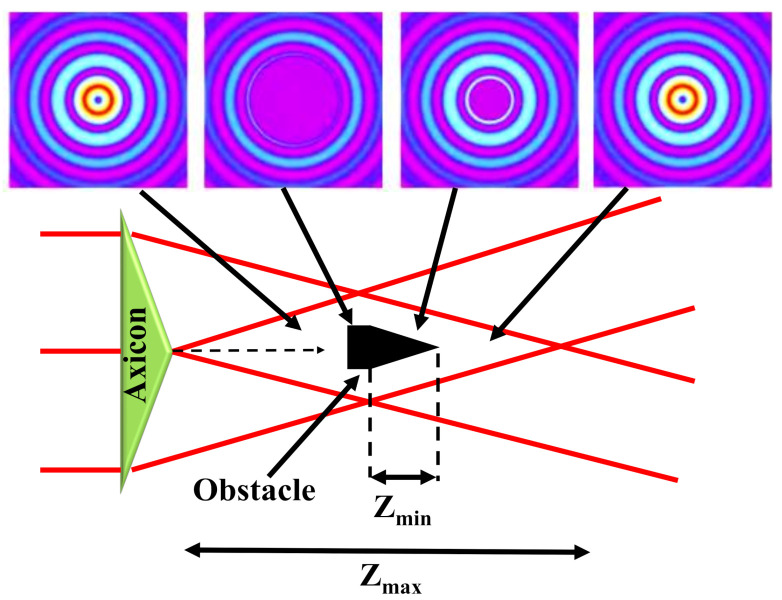
Self-healing features of an axicon-produced BB: an obstacle located in the center of the Bessel region blocks the beam for the smallest distance *Z_min_*, the Bessel field reconstructs after travelling for some distance. The beam profiles before and after the obstacle are also shown. Inspired by [120].

In addition to the non-diffractive feature, BBs are self-healing which means that they have a self-reconstruction ability after a hurdle comes across their transmission path [118]. The intensity arrangement at *z* and *z + δ_z_* is formed by different sections of the crossing planar beams as shown in Figure 6. This property comes from the selective constructive interference occurring between multiple coherent plane waves propagating at an equal angle concerning the optical axis. The redevelopment distance of the beam relies on the size of the hurdle and the angle defining the conical superposition of the planar beams. Consequently, BBs are usually more prone to dispersion than most other traditional Gaussian beams. Due to the unusual non-diffractive and self-recovery properties, BBs have gathered particular attention in biomedical physics, laser processing and metrology [119].

A novel method for creating long-distance self-recovering BBs generated by an annular lens and a 4f-configuration spherical lens is stated in [121]. This shows the diffraction-free light evolution of a zeroth-order BBs over several meters and addresses the available scaling prospects that exceed present technologies. Additionally, it has been shown that this setup can be modified to generate BB superpositions, realizing the longest optical conveyor beam and helicon beam. Last but not least, the self-healing properties of the beams are verified concerning robust opaque and transparent scatterers, which underscores the pronounced perspective of this novel approach [121].

Underwater wireless communication is supposed to have high data rates to relay big data over several meters via a proper wavelength. It has already gained more and more recognition with a massive rise in underwater applications, for instance, unmanned underwater vehicles, submarines and sensors in the oceanic environment [122,123,124], among others. The optical turbulence is primarily caused by variations in temperature and salinity in underwater environments, such as in oceans. The standard of communication of the orbital angular momentum (OAM)-based underwater wireless communication system has been seriously impaired by this turbulence [125]. A hypothetical analysis of the effect of temperature and salinity variations on the typical strength of the Gaussian Schell-model vortex beams in the turbulent ocean has shown that partially coherent beams have a more effective resistance to turbulence than full coherent beams [126]. The transmission of partially coherent Laguerre-GBs in the tempestuous ocean is studied in [127,128]. Furthermore, the influence of the ocean instabilities on the oceanic OAM-based underwater wireless communication system’s channel capacity has also been observed in [129]. In [126] the statistical characteristics of the vortex beams such as the power, polarization, and coherence travelling in oceanic turbulence are theoretically studied.

Vortex beams bearing OAM with helical phase fronts have been commonly used as beam sources to mitigate the turbulence effect on any optical vortex [130]. A Bessel-Gaussian (BG) beam is an imperative part of the pseudo-non diffraction vortex beams family. In the case of blockades, BG beams can heal themselves, which is an important feature for optical communications based on the line-of-sight operations [131]. Consequently, for the OAM-based underwater communication systems [132,133], the non-diffraction and self-recovery properties of BG beams make them a valuable commodity. The transmission characteristics of BG beams in the free space environment was explored [134] and the non-obstruction characteristics of BG beams in a free-space optical communication system were studied in [135].

**Figure 7:**
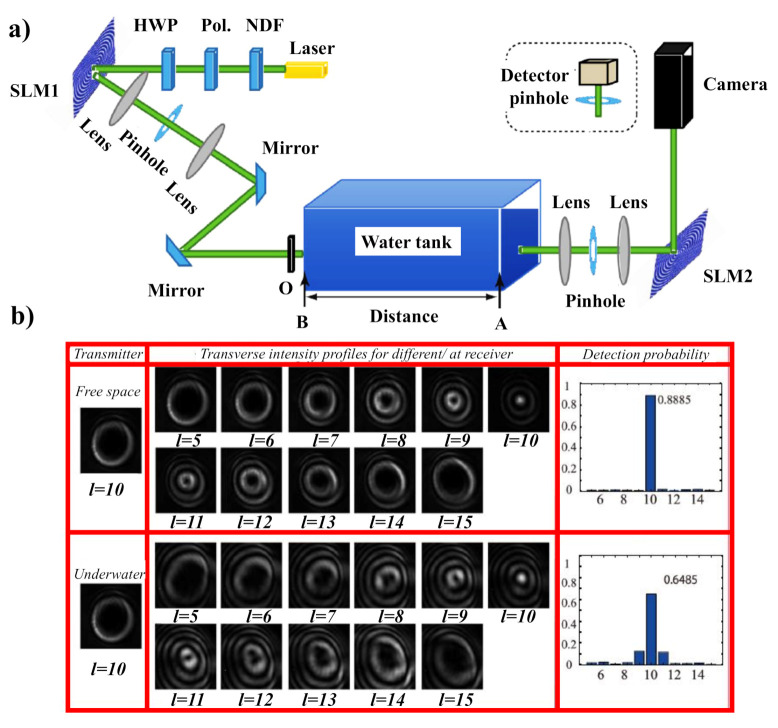
(**a**) The investigational arrangement for examining BG beam properties in the underwater situation. (**b**) The influence of underwater optical turbulence on the transmission of BG beam versus free space atmosphere. Adapted with permission from [131].

An experimental study of the underwater transmission and self-recovering properties of BG beams has recently been provided in [131]. A GB with a wavelength of 532 nm is transmitted from a laser diode and then a neutral density filter (NDF) is employed to reduce the intensity of the GB. The GB matches its polarization to the optimized working polarization of the selected polarization-sensitive spatial light modulator (SLM) after passing through the polarizer (Pol.) and a half-wave plate (HWP). When the polarized GB is illuminated on an SLM, the desired BG mode is generated, where the special phase hologram grating is revealed on the liquid crystal screen of the SLM. The BG beam carrying the OAM mode is then transferred through a water tank simulating the underwater environment. A rectangular water tank of one meter in length was used to replicate the underwater atmosphere. The temperature variations were regulated by a heater within the container, and salinity variations were accompanied by adding different salt bulks in the water. The experimental setup to analyze the BG beam properties underwater is shown in Figure 7a, whereas the recorded beam profiles at the receiver are shown in Figure 7b. For more detailed information, discuss [131].

## 5. BB for Optical Coherence Tomography

Optical coherence tomography (OCT) is an imaging process that offers in situ, non-invasive, high resolution, cross-sectional pictures of the biological tissues. Given the depth of penetration of a light beam into biological tissue, endoscopic probes are needed to examine the conditions of wall linings inside the organs such as the liver, stomach, lung, colon and wider arteries within the body. Various types of endoscopic OCT systems have been developed after the first endoscopic applications of OCT in 1996 [136]. Traditional GBs are currently used in endoscopic probes. Some are relocated to the targeted site by moving a wire backwards and forward in and out of the body to steer the catheter. These probe types work at the low numerical aperture and have a lateral resolution of ~20 μm. Some probes use higher numerical aperture optics; nevertheless, they are limited to the smaller DOF. A fine focus modification is needed for these probes which cannot be accomplished by wire adjustment. A gradient index (GRIN) lens rod-based probe was then demonstrated to focus on targeted points in a sample by shifting a stage out of the body [137]. Nevertheless, some endoscopic applications are constrained by the nonflexible rod. The growth of elongated DOF imaging arrangements is therefore an active research area of OCT [138]. Several methods have been applied, such as the Swept-source, time and spectral-domain OCT. Of these methods, the spectral-domain is favored over others, since it is simple to implement and does not require a complex structure. Initially, the OCT system was arranged only in free optics, but the advent of optical fiber improves the versatility of the system and paves the way for a new area of operation [139]. In OCT, the sample beam interferes with the reference beam to produce interference spectra that are further analyzed by the dedicated sample imaging tools. OCT may be achieved by either dual-path or a common-path interferometry techniques. The later methodology can solve the problems of polarization mismatch, group velocity dispersion and ambience vibration. The common path configuration eliminates device and computational complexity since there is no need for extra modules and algorithms to compensate for dispersion.

Imaging based on BB, produced by axicon optics, differs from GB imaging in that it allows the focusing range to be extended without losing resolution [140]. However, the trade-off is a reduction in the illumination and collection efficiency. OCT employing BB has become a trend to benefit from a long DOF and tighter spot size. Bessel beams for illumination/detection were used to break the limit of focus range and transversal resolution given by the GB profile [141,142,143]. The use of axicon optics in time-domain OCT was originally considered by Ding et al. [144] Lately, a non-dual path imaging scheme for imaging of biological samples has been suggested employing Fourier-domain OCT (FD-OCT) [145]. A custom-made micro-optic axicon is used to produce a BB to lighten and image in biological samples using spectral-domain OCT (SD-OCT) [146]. Concerning the GB, BB concurrently produces a high DOF and a tight focus point. It is not possible to produce perfect BB, but the GB can be converted into a BB structure known as a Bessel-Gauss beam and is often referred to as a BB. Several methods are discussed to transform the standard GB to BB. Traditionally, the axicon lens may be the best alternative for the transformation of the beam. A lot of impressive work has been performed on the generation of BB via the axicon lens [147,148]. However, BB axicon lens construction uses free optics, which are not appropriate for applications where there is a need for a small central spot size.

**Figure 8:**
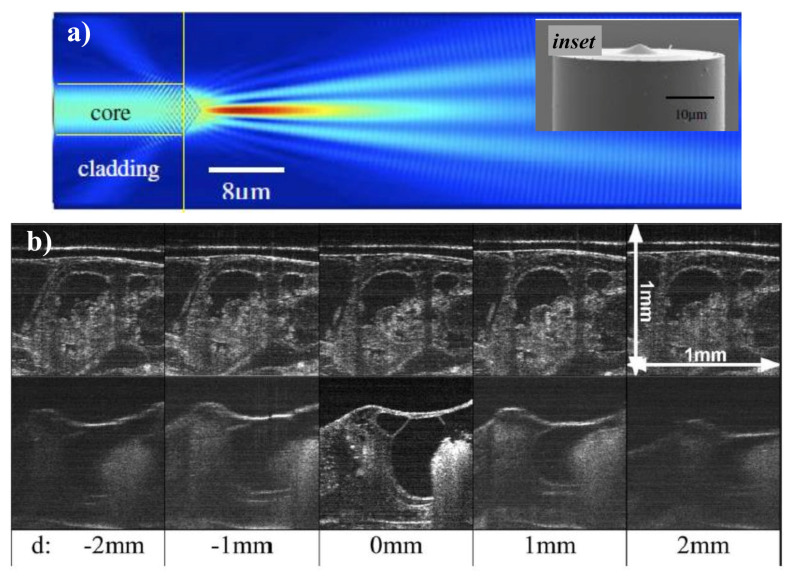
(**a**) Illustration of a light field travelling from the fiber to the sample and back. The inset displays the SEM image of the fiber probe where the conical microlens can be seen. Adapted with permission from [149], (**b**) 1 mm × 1 mm SD-OCT images of an African from tadpole located at various d values acquired using a 600 μm effective axicon micro-optic lens (top row) and a 0.037 NA conventional lens (bottom row). Adapted with permission from [146].

To eliminate group velocity dispersion and polarization incompatibility between the reference and the sample arm, common-path optical coherence tomography (CP-OCT) is used so that both arms share the same physical path. Current CP-OCT implementations usually involve one to incorporate an additional cover glass into the beam path of the sample arm to provide a reference signal. This step is further reduced by making direct use of the back-reflected signal, provided by the conical lens-tip fiber, as a reference signal as shown in Figure 8a [149]. The conical lens, which is directly manufactured by a simple selective-chemical etching process on the optical fiber tip (inset of Figure 8a), performs two functions: (1) it can be used as an imaging lens; (2) as the self-aligning reference plane.

Researchers have been involved in producing such axicon structures within the optical fiber such that they can be used for imaging and sensing applications. Considerable work is being conducted on BB construction using optical fibers and deep-sealed negative axicons (DSNAs) [150,151]. The DSNA is manufactured by standard chemical etching in hydrofluoric acid (HF) at the tip of the single-mode highly doped photosensitive optical fiber and produces BB [152]. A tomography methodology for fluorescence microscopy scanning is introduced in [153], which enables the volume image to be captured in a single frame scan. Volumes are photographed by concurrently recording four independent projections at various angles using temporally multiplexed, tilted BBs.

An FD-OCT method is used to exhibit the effectiveness of this method upon biological tissue. An in-fiber CP-OCT technique can prove to be potentially useful in endoscopic OCT imaging. The usefulness of employing axicon micro-optics has been solely established by imaging a biological sample, precisely an African frog tadpole, at different places attained with an incident power of 25 mW and an irradiation time of 50 μs as shown in Figure 8b The detailed study on SD-OCT can be found here [146].

## 6. Birefringence Detection Based on Astigmatic Transformed BB

**Figure 9:**
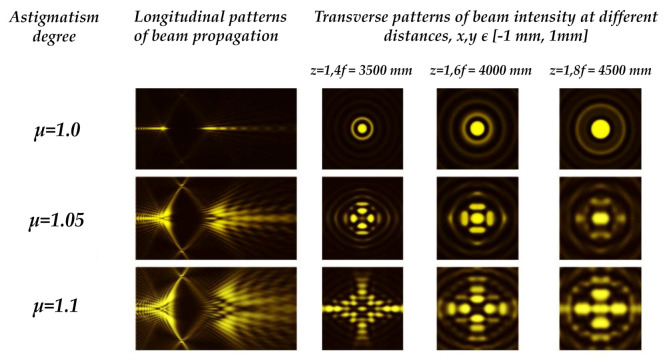
Birefringence detection established on the astigmatic transformation of a BB. Adapted with permission from [61].

One of the difficulties of information-measuring optical fiber networks is the random birefringence of the basic elements, for instance, the birefringence of a single-mode fiber (SMF) due to the ellipticity of the fiber core. Birefringence results in an unregulated change in the polarization of light, the properties of the optical fiber sensors and the values of the measured signals. Birefringence is often intrinsic in the GRIN owing to the residual mechanical stress led by the dopant, which results in a decrease in the performance of the radiation coupling into an optical fiber because of astigmatic misrepresentation and variations in the polarization state of the signal [154,155,156,157]. Birefringence control is therefore an essential practical task which provides a method for enhancing the correctness and consistency of transmitted signals in optic fiber and optoelectronic systems. In [61], Khonina et al. have proposed a mechanism for the detection of birefringence parabolic GRIN lenses by utilizing the astigmatic transformation of the zeroth-order BB as shown in Figure 9 [59,60]. Numerical modelling reveals a distinctly noticeable deformity of the intensity distribution in the transmission of scalable astigmatic BBs. The strength of astigmatism can be calculated by the electric field distribution of the distorted beam. In an experimental analysis of the propagation of a BB utilizing a quarter-pitch GRIN lens, similar patterns are also obtained, which suggests the existence of birefringence of the lens material. The assessment of the experimental and computational results indicates the probability of calculating an optical path difference between the ordinary and extraordinary beams of no worse than 0.05 of the wavelength.

**Figure 10:**
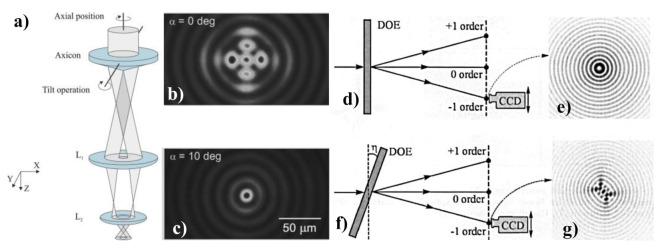
(**a**) BB generation setup, (**b**) Axicon generated BB pattern in the XY plane for 0 degrees, (**c**) Axicon-generated BB patterns in the XY plane for 10 degrees [158]. Optical setup for the generation of BBs at different diffraction orders employing a multi-order DOE under (**d**) perpendicular and (**f**) oblique illumination. This results in the intensity distribution for a perpendicular diffraction order under (**e**) perpendicular and (**g**) oblique illumination.

Besides, the possibility of optimizing the method of glass dicing by regulating the axicon-produced BB ellipticity (astigmatic) is proposed in [158]. In Figure 10a–c, the BB generation setup and the generated BB patterns are shown for the axicon tilt angle of 0 and 10 degrees. Intra-volume modifications are created by the astigmatic BB with transverse crack proliferation in the dominant direction. Regarding processing time, glass breaking force and cutting standard, the alignment of these adjustments parallel to the dicing path provides major benefits. A similar approach has also been demonstrated where five BBs of complex amplitudes was fabricated to study the astigmatic BBs. The optical setup and the BB patterns are shown in Figure 10d–g. The detail of this method can be found in [46].

## 7. Tight or Sharp Focusing and Focal Shaping

### 7.1. Superresolution

**Figure 11:**
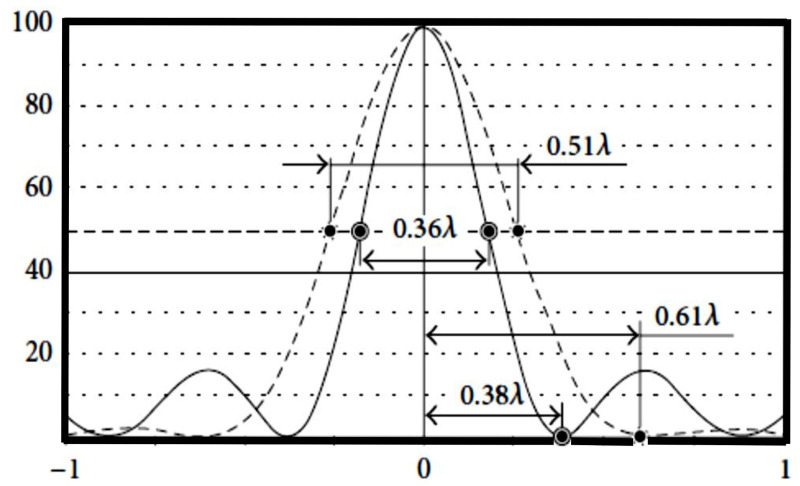
Drawing of the focal spot for the full-aperture radiance by plane wave—classical diffraction limit is 0.51λ and with narrow ring aperture—BB’s diffraction limit is 0.36λ (superresolution). Adapted with permission from [70].

A narrow annular pupil that blocks the light from propagating practically through the entire central part of the lens [70,159,160,161] is a simple, albeit low-efficiency technique to create narrow extended beams in the focal plane. From Figure 11, it can be seen that by establishing the narrow ring the generation of 1.6 times smaller focal spot even at the high numerical aperture is permitted. The detailed analysis of this topic can be found in our previous study [70].

More complex methods for the full-aperture apodization of the pupil’s function that utilizes both purely the phase and amplitude-phase distributions have also been presented [67,162,163]. One method is based on an additional axicon-type binary phase that discards the central rays from the focal plane [164]. This permits the reduction of the spot size in the total intensity with a reasonable loss of energy. However, in all cases, a tighter focal spot is usually attained at expense of the energy redeployment from the central peak to the side lobes.

### 7.2. Strengthening the Longitudinal Component

**Figure 12:**
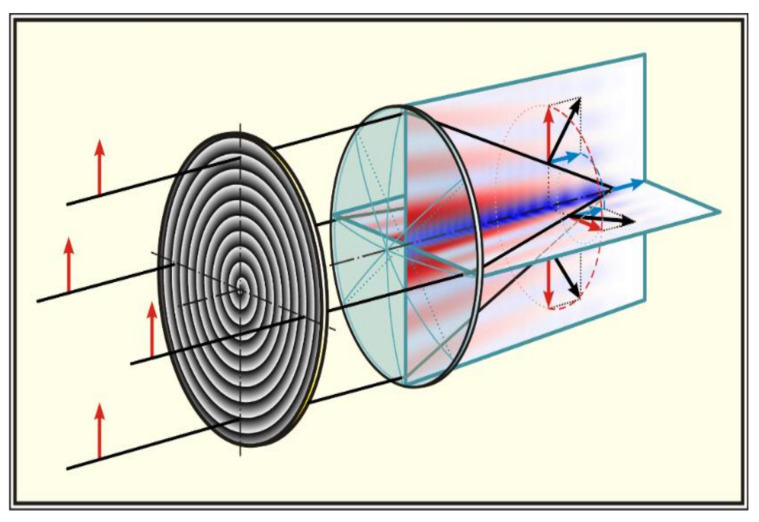
Generation a longitudinally polarized needle when a spiral diffractive axicon is added to the objective.

Note that the superresolution is also achieved due to special types of polarization and amplification of the longitudinal component of the electric field [165,166,167,168]. The longitudinal component plays a significant role not only in the reduction of the focal-spot size, but also in other applications, in particular, in the imaging of molecule orientations [169], second-harmonic generation [170], spectroscopy [171] and particle acceleration [172]. To generate and amplify the longitudinal component of the electric field, it is possible to use the optical elements, as well as the specially designed optimized elements that form longitudinally polarized needles as shown in Figure 12 [68,173,174,175,176].

**Figure 13:**
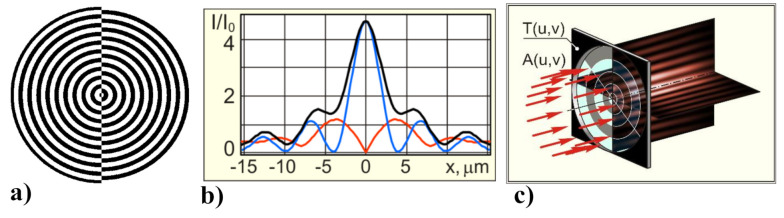
Asymmetric binary axicon (**a**) for the formation of a longitudinally polarized field, (**b**) the red line is for transverse components intensity, the blue line is for the longitudinal component intensity, and the black line is for the total (sum) intensity with linear polarization of the incident radiation, (**c**) Illustration of Scheme.

Note that a longitudinally polarized field can be formed immediately near the optical element using a large-aperture axicon [66]. Moreover, the enhancement of the longitudinal component is possible for various types of polarization due to the introduction of asymmetry into the axicon structure [69,177] as shown in Figure 13. 

### 7.3. 3D Focal Shaping

Shaping and transformation of light beams in the focal domain [178,179,180,181,182] is of paramount and growing interest in modern optics, such as in confocal microscopy, stimulated emission depletion, darkfield microscopy as well as in optical tweezers/particle trapping, optical storage, lithography etc. A chain of nearly spherical balls of subwavelength size is easy and can be formed by counter-propagating Bessel beams [183,184]. To form a separate spherical distribution, the interference of tightly focused vortex beams can be applied [185,186].

## 8. Other Related Applications

A lens is the best-known optical element, an axicon has become well known in optics only since the middle of the past century [140]. By an axicon was understood any axisymmetric optical element which, due to reflection and/or refraction, converts the light of a point source disposed on the optical axis into an axial segment [140]. Later, the term of the classical axicon began to be used to refer to an optical element whose phase function has a linear dependence on the radius—a linear or conical axicon [187,188]. At the same time, various axisymmetric optical elements have been proposed a logarithmic axicon [189,190], a generalized axicon [191], which generates an axial light segment with definite properties.

When an axicon is combined with a spiral phase plate, a spiral or helical axicon can be obtained [192]. For the first time, a diffractive spiral axicon was manufactured using the photolithography technology and was experimentally used to form higher-order BBs [193]. In the binary version, the spiral axicon looks like a spiral ring grating [194]. When an axicon is supplemented with a lens, another interesting optical element is obtained—a lensacon [195,196]—which was suggested to use a corrective element in the optical system of the human eye [197,198]. An artificial eye lens in the form of a thin plate whose surfaces have microrelief in the form of a circular diffraction grating can be implanted through a small incision, which significantly reduces surgery risks. An axicon supplemented by a lens generates a light ring [199,200], which is useful in surgery for smoothing and ablating corneal tissue [201].

Optical communication systems have been a popular tool for transmitting information due to high reliability and high usability. Many applications for personal, commercial and military use have been described. Owing to the high prevalence and diversity of applications, an optical network must be sufficiently protected. The protection applies to all levels of the network. It is also important to have the highest degree of protection on all layers. A very common and dangerous practice is to implement security protocols at higher levels of the network without protecting the lowest levels. To create a complicated solution, the physical layer of an optical system must be secured against different kinds of hazards. The physical layer of an optical fiber network is vulnerable to a range of threats, ranging from threats on physical networks, surveillance and eavesdropping.

**Figure 14:**
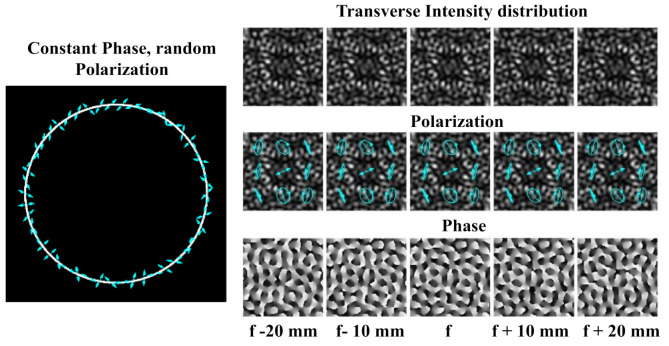
Transverse distributions of intensity, polarization and phase at different positions behind the lens for random polarization state. Adapted with permission from [65].

Non-diffractive beams are suitable for information encryption in optical communication because the diffraction-free property of the beams does not rely on the distribution of the amplitude, phase or even polarization of the spatial frequency range [62,63,64,202]. In [203], an experimental technique is described for easily generating a non-diffracting range of intensity patterns. It is expected that these patterns might be useful in information encryption or surveillance applications. Polarization state encryption has been shown to offer additional consistency in key encryption architecture, complementing amplitude and phase encryption [65,204,205,206]. Moreover, some recent studies have looked at the use of partially coherent light for optical trapping [207,208] and polarization-multiplexing for optical recording [209], making it feasible for related applications of transversely random polarization non-diffracting beams. Figure 14 presents the intensity, polarization and phase distribution for the constant phase and randomly polarized input beam.

**Figure 15:**
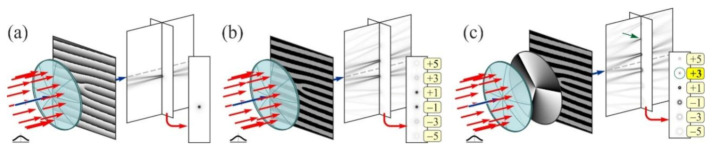
The action of the conventional fork grating with *m* = 1: (**a**) grayscale element, (**b**) binary element and (**c**) binary element in the presence of a vortex beam with *m*_0_ = 3. The focal plane is separately shown and the numbers in the frame resemble the values (nm), i.e., positive diffraction orders are positioned below. Adapted with permission from [220].

Lately, the researchers are attracted by the “perfect” optical vortices (POV) having ring radius independent of its vortex number [210,211,212,213]. As a rule, the Fourier transformation of BBs or lens-axicon doublets [213,214,215,216] is used to create such optical beams. In reality, these methods can be considered similar, since the axicon is regularly employed to produce BBs [147,217,218]. Besides, the POV can be shaped using a curved fork grating [219,220]. The operation of curved fork gratings are significantly different from classic fork gratings [221,222,223], which are used to generate and also to detect numerous OV beams having different diffraction orders [224]. With the creation of a correlation peak in the focal plane of the lens in the corresponding diffraction order, the existence of a vortex component of a specific order is observed (Figure 15).

**Figure 16:**
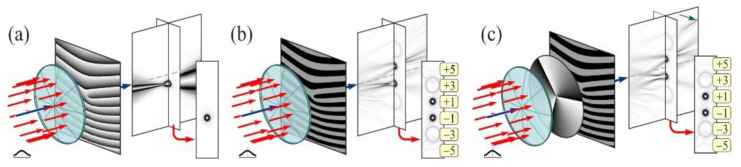
The action of the curved fork grating with *m* = 1 (**a**, **b**, **c** are analogical as Figure 15). Adapted with permission from [220].

Curved fork gratings generate multiplexed BBs, so annular distributions independent of its vortex number are formed in the focal plane. Hence, the detection of an OV cannot be comprehended in the focal plane, but only at a certain distance from it (see Figure 16). This offers additional security for information transmission.

Furthermore, the generation of a 3-D configuration of ring-shaped OV beams is important for the processing of high-throughput laser material. For example, the shaping of the desired configuration of OV beams enables the processing of a bulk of transparent materials and polymers and the manufacture of 3-D meta-structures using single-shot pulse laser printing [225].

## 9. Concluding Remarks

The optical beams free of dispersion and diffraction are exceptional cases of optical wave transmission that have gained extensive interest in recent years. The beams that are formed with spatial or temporal invariance involves a detailed knowledge of the physical laws governing their propagation in a given medium. A Bessel beam (BB) gets its name from the narrative of such a beam via a Bessel function, and this results in an anticipated cross-sectional contour of a set of concentric rings. Mathematically the BB contains an endless number of rings, and so over an infinite area would transfer infinite power. Thus the assumption must be that BB cannot be created. However, Durnin et al. demonstrated that an approximation can be made to a BB (quasi-BB) experimentally which retains the properties of the mathematical solution over a limited distance. The diffraction-free character of perfect BBs has initiated massive interest among researchers all over the world. In this review paper, we tried our best to enclose this vast topic so that the readers can find recent developments on BBs in one place. Moreover, we have also included several theoretical and experimental demonstrations of BBs from our research group.
